# Exploring Poly(ethylene glycol)-Polyzwitterion Diblock Copolymers as Biocompatible Smart Macrosurfactants Featuring UCST-Phase Behavior in Normal Saline Solution

**DOI:** 10.3390/polym10030325

**Published:** 2018-03-15

**Authors:** Noverra M. Nizardo, Dirk Schanzenbach, Eric Schönemann, André Laschewsky

**Affiliations:** 1 University of Potsdam, Institute of Chemistry, Karl-Liebknecht-Str. 24–25, D-14476 Potsdam-Golm, Germany; nizardo@uni-potsdam.de (N.M.N.); dschanz@uni-potsdam.de (D.S.); eschoenemann@uni-potsdam.de (E.S.); 2 Fraunhofer Institute of Applied Polymer Research IAP, Geiselberg-Str. 69, D-14476 Potsdam-Golm, Germany

**Keywords:** block copolymer, amphiphile, macrosurfactant, thermoresponsive self-assembly, polyzwitterion, upper critical solution temperature (UCST), salting-in

## Abstract

Nonionic-zwitterionic diblock copolymers are designed to feature a coil-to-globule collapse transition with an upper critical solution temperature (UCST) in aqueous media, including physiological saline solution. The block copolymers that combine presumably highly biocompatible blocks are synthesized by chain extension of a poly(ethylene glycol) (PEG) macroinitiator via atom transfer radical polymerization (ATRP) of sulfobetaine and sulfabetaine methacrylates. Their thermoresponsive behavior is studied by variable temperature turbidimetry and ^1^H NMR spectroscopy. While the polymers with polysulfobetaine blocks exhibit phase transitions in the physiologically interesting window of 30–50 °C only in pure aqueous solution, the polymers bearing polysulfabetaine blocks enabled phase transitions only in physiological saline solution. By copolymerizing a pair of structurally closely related sulfo- and sulfabetaine monomers, thermoresponsive behavior can be implemented in aqueous solutions of both low and high salinity. Surprisingly, the presence of the PEG blocks can affect the UCST-transitions of the polyzwitterions notably. In specific cases, this results in “schizophrenic” thermoresponsive behavior displaying simultaneously an UCST and an LCST (lower critical solution temperature) transition. Exploratory experiments on the UCST-transition triggered the encapsulation and release of various solvatochromic fluorescent dyes as model “cargos” failed, apparently due to the poor affinity even of charged organic compounds to the collapsed state of the polyzwitterions.

## 1. Introduction

**Scheme 1:**
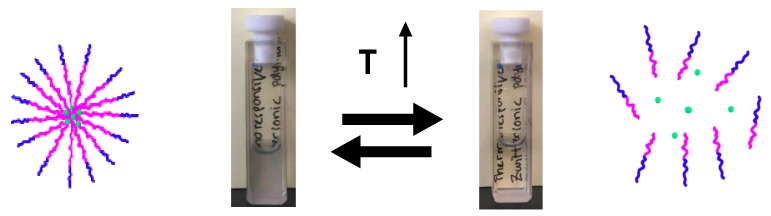
Idealized view of the temperature-induced micellar assembly/disassembly of a thermoresponsive block copolymer induced by heating through an UCST-type transition, and the concomitant release of cargo molecules (•) that are solubilized inside of the micellar aggregate at elevated temperature.

Amphiphilic block copolymers are generally considered to behave as “macrosurfactants” [1,2,3,4,5]. Despite their structural similarity to standard surfactants, they feature a distinct property profile with characteristic differences to their low molar mass counterparts. In particular, their self-assembly into micelles—or other aggregates, such as vesicles—is non-ergodic when the hydrophobic block exceeds a quite small critical size [6,7,8,9,10]: in this case, amphiphilic block copolymers require special preparation procedures for their self-assembly. Typically, the block copolymer is dissolved in a non-selective organic solvent that is water-miscible, the polymer solution is mixed with an excess of water, and finally, the solvent is removed, e.g., by evaporation or by dialysis [1,4,6,11,12]. To circumvent such a cumbersome procedure, “smart” macrosurfactants may be used, in which the hydrophobic block is designed to be stimulus-responsive and can reversibly change its character from hydrophilic to hydrophobic upon exposure to a trigger [8,13,14,15]. In this way, the amphiphilic character, and thus the self-assembly of the block copolymers, can be deliberately switched “on” and “off”. Furthermore, such a responsive aggregation behavior enables control of the system’s viscosity [16,17,18,19], as well as the transport and controlled delivery of poorly water-soluble active agents [20,21,22] (Scheme 1). 

Though pH-responsive polymers have been most commonly employed in practice until now, thermoresponsive systems have been increasingly explored as they allow for use in closed systems as well as under rather mild conditions. This seems to qualify them in particular for applications in fields such as cosmetics, personal care, biotechnology and biomedicine [21,22,23,24,25,26,27]. Obviously in such a context, the use of highly biocompatible polymers is preferable [22,28]. 

Two types of thermoresponsive polymers can be distinguished. The first is characterized by a phase diagram exhibiting a soluble-insoluble (“coil-to-globule” or “collapse”) transition with decreasing temperature, with an upper critical solution temperature (UCST). Based on simple thermodynamic considerations, such phase behavior would generally be expected, yet it is the exception for aqueous solutions of polymers [24,29,30,31,32]. The second type is characterized by a phase diagram exhibiting a collapse transition with increasing temperature and showing a lower critical solution temperature (LCST). While such phase behavior is not self-evident based on simple thermodynamic considerations, it is encountered for a plethora of non-ionic polymers in aqueous systems [15,30,33]. Accordingly, the vast majority of studies on thermoresponsive amphiphilic block copolymers has dealt with LCST-based systems. These have the additional advantage that their phase transition temperature—often determined by turbidimetry and thus synonymously referred to as cloud point (CP_LCST_) due to the clouding of the solution observed when phase separation sets in—can be easily tuned to any value for a given system. This can be not only achieved by adapting the chemical structure of a thermoresponsive homopolymer [30,33,34], but for instance also by (statistical) copolymerization [35,36,37,38], by (partial) chemical modification of a precursor polymer [39,40,41,42], or by anchoring particular end groups [43,44,45]. In the context of biocompatible polymers exhibiting an LCST transition in water, derivatives of poly(ethylene oxide) (PEO, or “poly(ethylene glycol)” PEG) play a most prominent role [20,28,36,46,47,48]. 

In contrast, UCST-based thermoresponsive block copolymer amphiphiles have rarely been investigated until now [32,49,50,51,52,53,54,55,56,57,58,59,60,61,62,63,64,65]. In fact, only a few polymers with UCST behavior in aqueous media have been described [29,66]. The majority of these is based on polyzwitterions bearing the sulfobetaine moiety that combines a quaternary ammonium cationic group with a sulfonate anionic group [67]. Importantly, this polymer class also seems to feature high biocompatibility [23,68,69]. Another particularity of poly(sulfobetaine)s is the high sensitivity of the UCST-transition temperature CP_UCST_ to the addition of low molar mass electrolytes, which typically dramatically enhances the water-solubility of the polymers (“salting-in” effect) [70,71,72,73,74,75,76]. UCST-based smart macrosurfactants seem particularly attractive for, e.g., controlled delivery purposes, as increased body temperature, caused by sickness, inflammation or otherwise enhanced metabolic activities etc., appears to be a much more useful trigger in biomedicine than reduced body temperature. Nevertheless, thermoresponsive macrosurfactants with UCST-behavior have only occasionally been reported, and studies on their use for controlled delivery have been exceptional so far [56,57,60,77]. 

**Figure 1:**
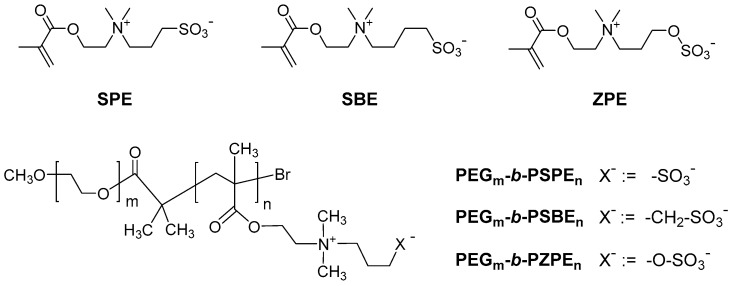
Structure of the zwitterionic monomers employed, and of the block copolymers synthesized and investigated. The number average degree of polymerization, DP_n_, of the PEG block was kept constant as *m* = 114. For the DP_n_ values *n* of the various polyzwitterion blocks, see “Results and Discussion”.

In the context of this general background, this study explores the synthesis and thermoresponsive behavior of block copolymers combining a non-ionic, a priori permanently hydrophilic PEG block with a polyzwitterion block which exhibits a UCST-type transition in aqueous solution (*cf.* Figure 1). This polymer design was also aimed at favoring high biocompatibility. An inherent difficulty of the chosen design is that while typical biological environments contain substantial amounts of salt, polyzwitterions are prone to pronounced salting-in effects. Consequently, we address the problem of implementing block copolymers that exhibit CP_UCST_ values in the physiologically most interesting temperature window of 30–50 °C at biologically relevant salinity. For our study, we used “normal saline solution” (NSS, also commonly denoted as ”physiological saline” or “isotonic saline”, i.e., 9 g·L^−1^ of NaCl, 0.154 M) as the model medium. Therefore, we selected for our study three polyzwitterions that exhibit a rather high UCST, or are even insoluble in pure water, expecting that the cloud points can be appropriately reduced in NSS. The explored polyzwitterion blocks comprise the well-established polysulfobetaine poly(3-((2-methacryloyloxyethyl)dimethylammonio)propane-1-sulfonate) PSPE, its rarely studied homologue poly(4-((2-methacryloyloxyethyl)dimethylammonio)butane-1-sulfonate) PSBE, and the structurally related poly(3-((2-methacryloyloxyethyl)dimethylammonio)propane-1-sulfate) PZPE (Figure 1). 

Bearing a sulfate instead of a sulfonate moiety as the anionic group, PZPE belongs to the class of polysulfabetaines of which only few examples have been reported so far, and that are notoriously poorly water soluble [78,79,80]. PSBE shows an UCST in pure H_2_O in the range of 80–100 °C for molar masses below 20 kg·mol^−1^, but is insoluble for higher ones [76]. While for PSPE, a number of conflicting values have been reported, clean polymers of sufficiently high molar mass seem to show an UCST of ca. 70 °C in pure H_2_O [76]. We explored the synthesis of these block copolymers via activators regenerated by electron transfer atom transfer radical polymerization (ARGET-ATRP) [81,82], and studied their thermoresponsive behavior in pure water as well as in normal saline solution (NSS).

## 2. Materials and Methods 

### 2.1. Materials

#### 2.1.1. Chemicals, Reagents and Solvents

The synthesis of cyanine dyes HC1 to HC4 has been described elsewhere [83,84]. Poly(ethylene glycol) methyl ether (“mPEG”, Fluka/Sigma Aldrich Schweiz, Buchs, Switzerland, *M*_n_ = 5000 g·mol^−1^) was dried by azeotropic distillation with toluene prior to use. Dansyl-l-phenylalanine (“DPA”, TCI, Deutschland GmbH, Eschborn, Germany, 98%), ethyl α-bromo isobutyrate (“EBiB”, Fluka, 98%), α-bromoisobutyryl bromide (Acros Organics/Fisher Scientific GmbH, Schwerte, Germany, 98%), 2-(dimethylamino)ethyl methacrylate (Sigma Aldrich, Chemie GmbH, Taufkirchen, Germany, ≥98%, stabilized by 2000 ppm hydroquinone monomethylether “MEHQ”), 1,3,2-dioxathiane 2,2-dioxide (“propylenesulfate”, TCI, ≥98%), triethylamine (Acros, 99%), dichloromethane (J. T. Baker, Phillipsburg, NJ, USA, 99.8%), diethyl ether (ChemSolute/Th. Geyer, Renningen, Germany, 99.8%), toluene (Merck Schuchard, Hohenbrunn, Germany, 99.8%), methanol (Avantor Performance Materials, Center Valley, PA, USA, 99.5%), trifluoroethanol (“TFE”, Carl Roth GmbH, Karlsruhe, Germany, 99.8%), 2,2′-bipyridyl (Fluka, 99%), pentamethyldiethylenetriamine ("PMDETA", Sigma-Aldrich, 99%), hexamethyltriethylenetetramine (“HMTETA”, Sigma-Aldrich, 97%), l-ascorbic acid 6-palmitate (Alfa Aesar, Karlsruhe, Germany, 95%), CuBr (Sigma-Aldrich, ≥97%,), NaCl (ChemSolute, Renningen, Germany, 99%), MgSO_4_ (Alfa Aesar, 99.5%), chloroform-d (Armar, Döttingen, Switzerland, CDCl_3_, 99.8 atom% D), D_2_O (VWR International GmbH, Darmstadt, Germany, 99.9 atom% D), molecular sieves 3 and 4 Å (Carl Roth GmbH) and cellulose dialysis membranes type ZelluTrans (Carl Roth GmbH), nominal cut-off MW 3500) were used as received. Acetonitrile (Carl Roth GmbH, ≥99.9%) was dried over CaH_2_. Deionized water was further purified by a Millipore Milli-Q Plus water purification system (Merck Millipore, Darmstadt, Germany, resistivity 18 MΩ·cm^−1^). 

#### 2.1.2. Monomers

The 3-((2-methacryloyloxyethyl)dimethylammonio)propane-1-sulfonate) SPE (Sigma-Aldrich) was used as received. The 4-((2-methacryloyloxyethyl)dimethylammonio)butane-1-sulfonate) SBE was kindly provided by Viet. Hildebrand; its synthesis is described in reference [76]. The 3-((2-methacryloyloxyethyl)dimethylammonio)propane-1-sulfate) ZPE was synthesized by adapting a previously reported procedure [80]. By modifying the recipe, the yield could be improved. Also, the amounts of highly toxic alkylating agent propylenesulfate remaining in the reaction mixture were strongly minimized thus facilitating the work up. In detail, 2-(dimethylamino)ethyl methacrylate (61.9 g, 394 mmol, 1.1 eq) in dry acetonitrile (50 mL) was added to a stirred solution of 1,3,2-dioxathiane 2,2-dioxide (49.5 g, 358 mmoL, 1.0 eq.) in dry acetonitrile (400 mL) at ambient temperature. The mixture was stirred at 50 °C for three days. When the mixture was cooled to ambient temperature, the monomer began to precipitate. Acetone was added to complete its precipitation. The solid was filtered off, washed with acetone, and dried in vacuo. Monomer ZPE was obtained as a colorless solid (102.8 g, 97%).

^1^H NMR (300 MHz, D_2_O, 298 K) δ (ppm) = 6.19 (s, 1H, CH=C–COO– (*cis*)), 5.81 (s, 1H, CH=C–COO– (*trans*)), 4.67 (s, 2H, –COO–CH_2_–), 4.19 (t, *J* = 5.6, 2H, –CH_2_–OSO_3_^−^), 3.89–3.80 (m, 2H, –COO–C–CH_2_–N^+^–), 3.68–3.56 (m, 2H, –N^+^–CH_2_–C–C–OSO_3_^−^), 3.24 (s, 6H, –N^+^–(CH_3_)_2_), 2.35–2.22 (m, 2H, –CH_2_–C–OSO_3_^−^), 1.97 (s, 3H, =C–CH_3_). ^13^C NMR (75 MHz, D_2_O, 298 K) δ (ppm) = 168.37 (–COO–), 135.11 (=C–COO–), 127.75 (=CH_2_), 65.29 (–N^+^–C–C–C–OSO_3_^−^), 62.68 (–COO–C–C–N^+^–), 62.38 (–N^+^–C–C–C–O–SO_3_^−^), 58.35 (COO–C–C–N^+^–), 51.28 (–N^+^–(CH_3_)_2_), 22.37 (–N^+^–C–C–C–O–SO_3_^−^), 17.30 (C–CH_3_). HR-MS (ESI): calculated: 296.1162 [M + H]^+^; found: 296.1167 [M + H]^+^. 

Elemental analysis (C_11_H_21_NO_6_S, *M*_r_ = 295.35): calculated: C = 44.73%, H = 7.17%, N = 4.74%, S = 10.85%; found: C = 44.65%, H = 6.87%, N = 4.82%, S = 11.40%. FT-IR (selected bands in cm^−1^): 3038 ν(N^+^–CH_3_), 2987 ν(CH_3_), 1715 ν(C=O), 1630 ν(C=C), 1156 ν_as_(SO_3_^−^), 1025 ν_s_(SO_3_^−^).

#### 2.1.3. Polymer Synthesis

The synthesis of macroinitiator poly(ethylene glycol) monomethyl ether 2-bromoisobutyrate (mPEG-Br) was adapted from the work of Ranger et al. [20]: At ambient temperature, 10.0 g (2.00 mmol) of dried poly(ethylene glycol) methyl ether (*M*_n_ = ~5000 g/mol) in 200 g of toluene were purged with nitrogen for 20 min to remove any oxygen, before 0.40 g (4.0 mmol) of triethylamine were added dropwise over 30 min under stirring. Then, 0.912 g (4.00 mmol) of α-bromoisobutyryl bromide were added dropwise over 1 h to the stirred mixture, and the reaction was allowed to proceed for 48 h. For the work up, the reaction mixture was extracted with water. The aqueous phase was thrice re-extracted with 50 mL of dichloromethane. The organic phases were combined, washed with neutral water, dried with MgSO_4_, and filtered through a filter paper. The majority of the solvent was removed by evaporation until a crude viscous extract was obtained, which was precipitated into diethyl ether. The colorless solid formed was isolated by filtration, and dried overnight in vacuo. Yield: 50%, colorless hygroscopic solid. ^1^H NMR (δ [ppm] in D_2_O): 1.94 (6H, –C(CH_3_)_2_Br), 3.48 (3H, –O–CH_3_), 3.5–4.0 (4H, –CH_2_–CH_2_–O–), 4.4 (2H, –COOCH_2_-).

If not otherwise specified, polymers were synthesized following a general procedure, by which the zwitterionic monomers were polymerized in TFE solution using either the low molar mass initiator EBiB, or for block copolymers, the macroinitiator mPEG-Br, CuBr with 2,2′-bipyridyl (bpy) as catalyst, and l-ascorbic acid 6-palmitate as reducing agent. 

In a typical procedure, 0.370 g (0.074 mmol) of mPEG-Br, 10.6 mg (0.074 mmol) of CuBr, 23.2 mg (0.148 mmol) of 2,2′-bipyridyl, and 15.4 mg (0.037 mmol) of L-ascorbic acid 6-palmitate were dissolved in 6 mL of TFE, and deoxygenated by bubbling nitrogen. This initiator-catalyst solution was transferred under a nitrogen blanket into the deoxygenated solution of 2.065 g (7.400 mmol) of monomer SPE in 6 mL of TFE. The ratio of [monomer]:[initiator]:[catalyst]:[ligand] was 100:1:1:2. Nitrogen was bubbled through the mixture for 30 min while stirring. Then, the mixture was placed into an oil bath at 60 °C. After 24 h, the reaction was stopped by cooling and exposing the solution to the air. Purification was done by dialysis in tubes against distilled water for five days exchanging the dialysate water at least once per day, and subsequent lyophilization to yield the solid polymer. 

### 2.2. Methods

Elemental analysis was carried out using a Vario ELIII micro analyzer (Elementar Analysensysteme, Hanau, Germany). High resolution mass spectra (HR-MS) were recorded with a mass spectrometer ESI-Q-TOFmicro (Quadrupol—Time of Flight, Thermo Fisher Scientific, Waltham, MA, USA). Electrospray ionization (ESI) using water as solvent was chosen as method. Fourier transform infra-red (FT-IR) spectra were recorded in a N_2_ purged atmosphere with a Thermo Nicolet Nexus FT-IR spectrometer (Thermo Fisher Scientific, Waltham, MA, USA) equipped with an attenuated total reflection (ATR) Smart Endurance element. ^1^H and ^13^C NMR spectra were recorded with a Bruker Avance 300 spectrometer (300 and 75 MHz, respectively, Bruker, Billerica, MA, USA) or with a Bruker Avance 400 spectrometer (400 and 125 MHz, respectively) at ambient temperature in deuterated solvents. Polyzwitterions were measured in 0.5 M NaCl in D_2_O, in which all samples formed clear solutions. ^13^C NMR spectra were recorded in ^1^H-broad band decoupling mode and in attached proton test (APT) mode, respectively. Solvent signals were used as internal shift references. 

Size exclusion chromatography (SEC) was performed with an apparatus SEC3010 (WGE—Dr. Bures, Dallgow-Döberitz, Germany) equipped with a refractive index detector, using PL-HFIPgel columns (Agilent Technologies, Santa Clara, CA, USA), hexafluoroisopranol (HFIP) containing 50 mM of sodium trifluoroacetate as eluent, and narrowly distributed poly(methyl methacrylate) standards (PMMA, PSS Polymer Standard Service, Mainz, Germany) for calibration.

Ultraviolet-visible (UV–Vis) absorption spectra were recorded by a UV/Vis/NIR spectrometer Lambda 19 instrument (Perkin Elmer, Waltham, MA, USA). Fluorescence spectra were recorded with fluorescence spectrometers FLS920-stm (Edinburgh Instruments, Livingston, UK) with the excitation and emission slits set to 1 nm, and FluoroMax-4 (Horiba Jobin Yvon, Bensheim, Germany) equipped with a thermostated cell holder, with slit widths of 2 nm. Various excitation wavelengths were used. In all cases, quartz cuvettes with an optical path length of 1 cm were used. 

Dynamic light scattering (DLS) was carried out with an instrument high performance particle Sizer (HPPS-5001, Malvern Instrument, Malvern, UK) using a He-Ne laser beam, and a thermoelectric Peltier element to control the temperature of the sample cell. The backscattering mode was used at a scattering angle of θ = 173°. Samples were prepared by dilution with Millipore water, or with NSS to the desired concentration.

Cloud point measurements used a Varian Cary 50 Scan UV–Vis spectrophotometer (Agilent, Waldbronn, Germany) equipped with a thermoelectric Peltier element for temperature control. In an optical silica cuvette of 1 cm inner path length, the transmission of polymer solutions was monitored at 500 nm as a function of temperature with cooling and heating rates of 1 K·min^−1^. The cloud points upon cooling, CP_UCST_, and upon heating, CP_LCST_, were defined as the temperatures where a sharp decrease of transmittance sets in. 

## 3. Results and Discussion

### 3.1. Polymer Synthesis

**Figure 2:**
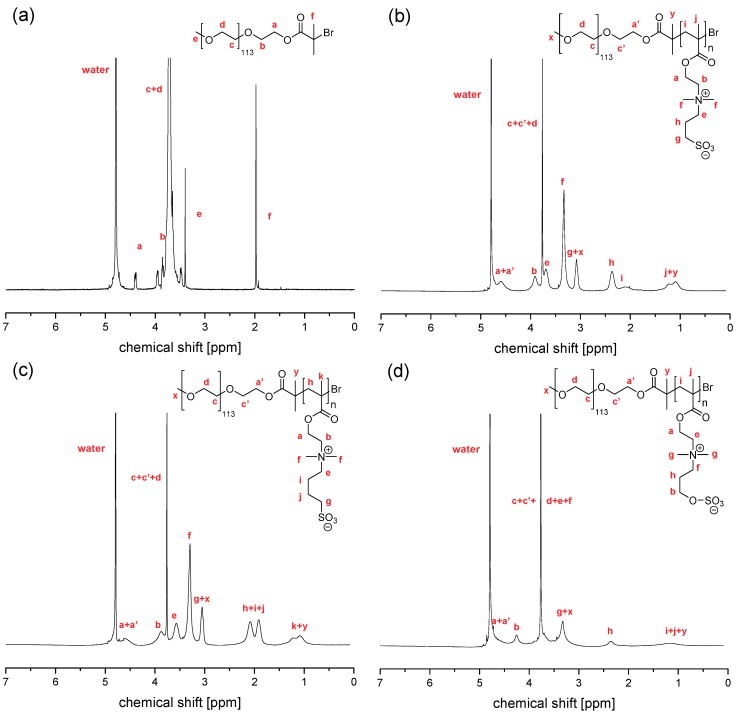
^1^H NMR spectra of (**a**) macroinitiator mPEG-Br in D_2_O, and of the derived block copolymers in 0.5 M aq NaCl: (**b**) mPEG-*b*-PSPE-6, (**c**) mPEG-*b*-PSBE-2, and (**d**) mPEG-*b*-PZPE-1.

The nonionic-zwitterionic block copolymers were prepared by ARGET ATRP polymerization of zwitterionic methacrylates (Figure S1), and characterized by standard methods such as ^1^H NMR and SEC. The integration of the ^1^H NMR signals of macroinitiator mPEG-Br (see Figure 2a) corroborates its number average molar mass M_n_ of 5.2 kg·mol^−1^ (DP_n_ = 114; using the integral ratios of signals “a”–”d” vs. “e”), as well as the quantitative esterification (using the integral ratios of signals “e” vs. “a” or “f”). The successful extension of the PEG block to create the zwitterionic copolymers PEG_114_-*b*-PSPE_n_, PEG_114_-*b*-PSBE_n_, and PEG_114_-*b*-PZPE_n_ (*cf.* Figure 1) is already qualitatively demonstrated by the ^1^H NMR spectra, which show the presence of both constitutional repeat units in the product (Figure 2b–d). This is corroborated by the SEC elugrams, which show that the molar mass distributions shifted to longer elution times compared to the macroinitiator employed, without an indication of any remaining unreacted macroinitiator. Polymerization conditions and results are summarized in Table 1.

The synthesis of block copolymers from methacrylate monomers by ATRP using PEG macroinitiators has been well established [20,85,86]. Exceptionally, even examples for PEG-containing block copolymers with polysulfobetaines have been reported [54,62,87]. Nevertheless, the synthesis of such block copolymers is not trivial. The polymerization of the zwitterionic monomers suffers from the need to use highly polar and protic solvents, such as trifluoroethanol TFE, when conducting the reaction in homogeneous phase [67,88]. Such solvents may interact or react with the copper catalyst system. Moreover, the high sensitivity of the aqueous solution phase behavior of polyzwitterions against even small amounts of electrolytes makes a thorough purification of the polymer products necessary to remove the transition metal catalysts that are inherently present. Accordingly, the proper combination of monomer, initiator, catalyst (both components, transition metal salt and ligand), solvent, and further optional additives has to be established.

Initial orienting experiments with SPE showed that copper-mediated ATRP of SPE in TFE—and also in aqueous methanol—worked sufficiently well with the classical ligand 2,2-bipyridyl (bpy). Block copolymers were readily accessible, and the theoretically expected number of average molar masses agreed well with those derived from the integration of the NMR spectra under the assumption that the molar mass of the incorporated PEG-block corresponds to that of the macroinitiator employed. The difference between the values of *M*_n_^theo^/*M*_n_^NMR^ and M_n_^app^ can be explained by the use of PMMA standards for calibrating the SEC elugrams. Still, we note that the dispersity values Ð tend to increase to 1.5 and higher for elevated molar masses. These rather high values of Ð compared to optimized ATRP systems might be blamed to difficulties with the SEC analysis (which is not trivial for zwitterionic block copolymers). Still, we rather suppose that they are mainly indicative of a limited control on the polymerization. Therefore, alternatively, we explored the use of other solvents such as aqueous methanol, or of ligands pentamethyldiethylenetriamine (PMDETA) and hexamethyltriethylenetetramine (HMTETA), but noticed no improvement. Also, the addition of *N*-alkylimidazolium chlorides, as suggested in the literature [89,90], did not reduce the Ð values (data not shown), while rendering the purification of the polymers even more cumbersome. We noted, however, that during the polymerization of ZPE in TFE, care must be taken to keep the reaction mixture dry. Otherwise, the sulfate moiety undergoes partial hydrolysis, which is possibly catalyzed by the Lewis acids Cu^+^ and/or Cu^2+^ present. 

**Figure 3:**
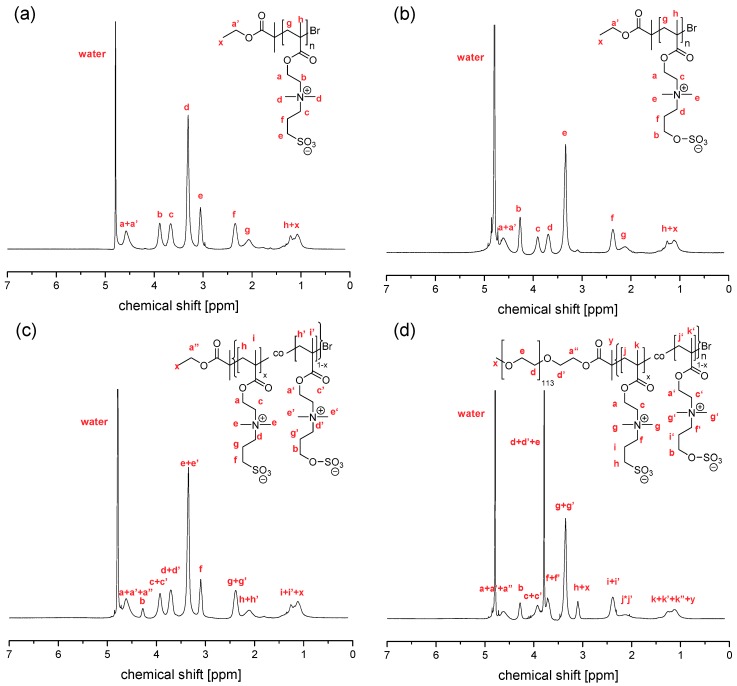
^1^H NMR spectra in 0.5 M aq NaCl (D_2_O) of (**a**) homopolymer PSPE-2, (**b**) homopolymer PZPE-1, (**c**) statistical copolymer P(SPE-*co*-ZPE)-1, and (**d**) statistical block copolymer mPEG-*b*-P(SPE-*co*-ZPE)-2.

As the water solubilities and the UCST values of the three parent polyzwitterions—PSPE, PSBE, and PZPE—were known to differ substantially (*vide supra*), we also investigated the use of their copolymers to enable the tuning of their CP values by the polymers’ chemical structure, not only by their molar mass (Figure 3, Table 2).

This strategy is well established for polymers featuring LCST behavior [35,36,37,38], yet difficult to implement for polyzwitterions, because it suffers from the poor compatibility and copolymerization behavior of the highly polar zwitterionic monomers with non-ionic ones. However, a priori, ideal azeotropic statistical copolymerization—i.e., identical reactivities of the comonomers employed—would be needed to obtain decently defined copolymer structures, also independently of the precise conversion. The chemical heterogeneity of the copolymers formed is detrimental to a well-defined “switching” behavior. This problem may be minimized by using particular solvents such as ionic liquids [91], which however render the work up and purification cumbersome. Also, the problem may be circumvented by post-polymerization modification strategies [42,49,74]. However, in turn, these pose problems with respect to the purity and reproducibility of the precise chemical structure obtained, for instance due to side reactions and incomplete conversion. Therefore, we addressed the copolymerization of sulfobetaine with sulfabetaine monomers, which bear the same polymerizable moiety and have very similar polarities (Figure 3 and Figure S1). 

Supposedly, this favors copolymerization reactivity ratios r_1_ and r_2_ of 1, and consequently, ideal azeotropic statistical copolymerization. The pair SPE and ZPE was chosen as their polymers present the extremes in the UCST-behavior of the three polyzwitterion systems studied. Moreover, the composition of their statistical copolymers can be readily analyzed via ^1^H NMR spectroscopy as illustrated in Figure 3, using signals “f” of the SPE units and signal “b” of the ZPE units (see Figure 3c). 

As for the binary block copolymers, the successful extension of the PEG block to give the zwitterionic statistical block copolymers PEG_114_-*b*-P(SPE-*co*-ZPE)_n_ is already qualitatively demonstrated by the ^1^H NMR spectra, which show the incorporation of both monomers in the products (Figure 3d), and by the SEC elugrams. These display molar mass distributions that are shifted to shorter elution times compared to the macroinitiator employed. The statistical copolymerization conditions and results are listed in Table 2. The comparison of the data in Table 1 and Table 2 demonstrates that the statistical copolymerization of binary copolymers as well as of statistical block copolymers worked out quite similarly to the reactions, in which one single monomer was employed. The reaction rates, molar masses and dispersities achieved are comparable. Hence, the strategy of copolymerizing structurally closely related zwitterionic monomers is a valid synthetic option. 

### 3.2. Thermoresponsive Behavior in Water and in Normal Saline Solution (NSS)

The aqueous phase behavior of the various block copolymers polymers was screened by turbidimetry, following the transmittance of an aqueous solution of a defined polymer concentration as a function of the temperature. All cloud point values were derived from cooling runs. Still, the hysteresis between heating and cooling runs was small (typically ≤ 1 °C). As expected [78,80], the zwitterionic polymers based on monomer ZPE were much more difficult to dissolve in water than the ones based on monomers SPE or SBE. Therefore, while the latter were characterized in 3 wt % solutions to facilitate comparisons with literature data [76], cloud point studies of the former were performed in more dilute aqueous solutions of 0.3 wt %. As data on the cloud points of PZPE and ZPE-based copolymers were not available, homopolymer and statistical copolymer samples of PZPE, P(SPE-*co*-ZPE) and also PSPE were studied additionally as references. Table 3 summarizes the cloud points determined. 

**Figure 4:**
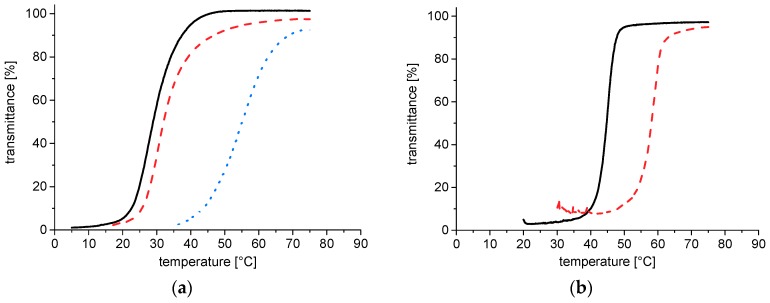
Temperature-dependent turbidity (cooling runs) of: (**a**) 30.0 g·L^−1^ aqueous solutions of SPE-based block polymers PEG-*b*-PSPE-3 (**—**), PEG-*b*-PSPE-4 (**- - - -**) and PEG-*b*-PSPE-5 (**- - - -**); (**b**) 3.00 g·L^−1^ solutions of PZPE homopolymers PZPE-1 (**—**) and PZPE-2 (**- - - -**) in NSS.

The first qualitative analysis of the data already revealed some key features of the phase transition behavior: (1) for a given degree of polymerization DP_n_ of the zwitterionic block, CP_UCST_ increases in the order PSPE < PSBE < PZPE, reflecting the behavior of the underlying homopolymers [76,80]; (2) within the molar mass range studied, CP_UCST_ values rise markedly with increasing DP_n_ (*cf.* Figure 4), also in agreement with the available data on the underlying homopolymers [76]; (3) while due to their extremely high CP_UCST_ values PEG-*b*-PZPE block copolymers do not seem appropriate for thermo-responsive systems in pure water, contrariwise, PEG-*b*-PSPE and even PEG-*b*-PSBE block copolymers seem of little use for thermo-responsive systems in NSS as the salting-in effect of NaCl suppresses at this concentratixon the UCST transition completely; (4) statistical copolymerization of SPE and ZPE allowed us to obtain CP_UCST_ values that are between those of the parent homopolymers PSPE and PZPE, and are suited for establishing thermo-responsive systems in both pure water as well as in NSS. 

Beyond these general findings, a closer look at the turbidimetric curves and the data in Table 3 reveals a number of interesting details. Apparently, the coupling to PEG of the block copolymers reduces the CP_UCST_ of the polysulfobetaines. Such an effect had been previously reported for other double hydrophilic block copolymers containing PSPE and PSBE [65], but the effects were weaker. Alternatively, the low value of CP_UCST_ = 23 °C for sample PSPE-1, and the outlier of PEG-*b*-PSPE-6 within the block copolymer series based on SPE correlating CP_UCST_ with DP_n_, may suggest that small amounts of salts from the catalyst system can remain in the polymers, thus artificially lowering the cloud points. In fact, poly(sulfobetaine)s are not only known to be very sensitive to salting-in effects, but also to stick tenaciously to many low molar mass salts [72,91]. Accordingly, the CP_UCST_ values measured in pure water have to be noted with some care, as they may represent only the lower limits of the true values. The CP_UCST_ values determined in NSS, however, seem realistic, as the high amount of NaCl present will cover such impurity effects. 

**Figure 5:**
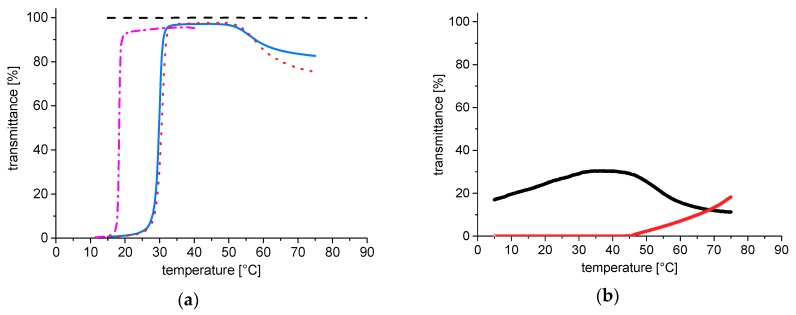
Temperature dependent turbidity of 30 g·L^−1^ aqueous solutions of sulfobetaine polymers: (**a**) PSPE-1 (- · - · -), mPEG-Br (**- - - -**), and 1:1 *w*/*w* mixture of PSPE-1 and mPEG-Br in cooling (**—**) or (**·····**) heating runs; (**b**) cooling runs of block copolymers PEG-*b*-PSBE-1 (**—**) and PEG-*b*-PSBE-2 (**—**).

**Scheme 2:**
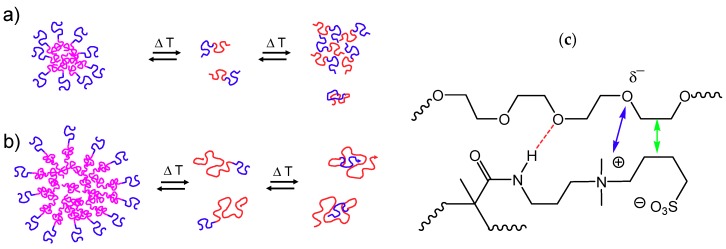
Hypothetical two-step, thermo-responsive self-assembly of block copolymers PEG-*b*-SBE. (**a**) if the zwitterionic and the nonionic blocks are of similar size, (**b**) if the zwitterionic block is much larger than the nonionic one, (**c**) possible interactions between the zwitterionic and the nonionic blocks: hydrogen bonding (**----**), electrostatic interactions (monopole—dipole ←→), hydrophobic (←→) interactions. In (**a**,**b**), lines signify: **—** = nonionic block, **—** = zwitterionic block below UCST-transition, **—** = zwitterionic block above UCST-transition.

Nonetheless, we wanted to check the possibility that specific interactions between the PEG block and the polysulfobetaine block might be responsible for the low cloud points found. To achieve this, we compared the clouding behavior of an aqueous solution of a mixture of PEG and PSPE homopolymers with the behavior of solutions of the two homopolymers alone (Figure 5a). While the pure PSPE showed an UCST-type transition and the pure PEG showed no thermal transition, as expected, the mixing of both homopolymers increased not only the CP_UCST_ by about 10 °C, but also induced an LCST-type phase transition around 55 °C. Accordingly, on the one hand, the often seemingly low CP_UCST_ values of the zwitterionic polymers in pure water suggest the presence of small salt impurities that could not be completely removed by the dialysis work up. On the other hand, the surprising behavior of the polymer mixture indicates marked interactions between both the non-ionic and the zwitterionic polymers (*cf.* Scheme 2c), which can potentially complicate the thermoresponsive behavior of their block copolymers. 

While we did not notice any particular behavior for the block copolymers bearing SPE blocks in pure water (*cf.* Figure 4a), some block copolymers bearing SBE blocks did indeed exhibit the superposition of an UCST-type and an LCST-type clouding transition (Figure 5b). This can result in so-called “schizophrenic” self-assembly, in which the roles of the insoluble block—which induces aggregation—and of the soluble block—which stabilizes the colloidal aggregate—are eventually interchanged [49], as reported previously for non-ionic–zwitterionic block copolymers of PSBE and poly(*N*-isopropylmethacrylamide), for instance [65]. In the rather narrow intermediate temperature range between the UCST- and the LCST-type transitions, the solution does not become completely clear. Similar behavior has been reported previously for other “schizophrenic” nonionic–zwitterionic diblock copolymers, and was attributed to concentration fluctuations [64]. Interestingly, we observed the simultaneous appearance of an UCST- and a LCST-transition only for PEG-*b*-PSBE-1, but not for the homolog PEG-*b*-PSBE-2. We can only speculate at present about this finding (*cf.* Scheme 2); possibly, the modulation of the self-assembly by the interaction of the PEG and the poly(sulfobetaine) blocks is sufficiently effective only when both blocks are of similar size, as it is the case for PEG-*b*-PSBE-1, but not for PEG-*b*-PSBE-2. 

In order to clarify the unusual clouding phenomena of PEG-*b*-PSBE-1, we also examined the aqueous solution by DLS as function of the temperature (Figure S3), but the findings were not conclusive. At 30–50 °C, rather broadly distributed aggregates with hydrodynamic radii *R*_h_ around 35 nm were observed, explaining the moderate turbidity observed in the intermediate temperatures range. The gradual decrease of transmittance upon cooling to 20 °C seems to result from a broadening of the size distribution, increasing the share of aggregates with larger radii before larger aggregates appear upon further cooling. At temperatures above 50 °C, the increased turbidity is the consequence of the disproportionation of the medium-sized aggregates into a mixture of small objects, possibly individual macromolecules, and very large aggregates of sizes up to 1 μm.

**Figure 6:**
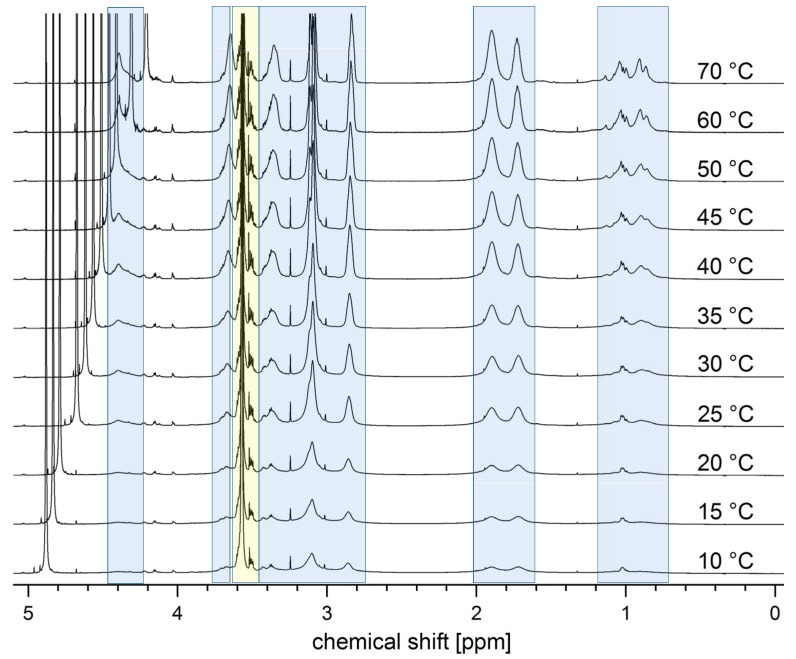
Temperature dependent ^1^H NMR spectra of PEG-*b*-PSBE-1 in D_2_O.

In order to learn more about the complex thermoresponsive behavior of PEG-*b*-PSBE-1, we followed the evolution of the NMR spectra in water in function of the temperature (Figure 6). Clearly, the signal characteristics of the PSPE block, which are highlighted in blue, are strongly broadened at low temperatures, but gain both intensity and at least moderate resolution upon heating beyond 35 °C. Although markedly attenuated, the PSBE signal characteristics for the 4-ammoniobutylsulfonate moiety (between 1.5 and 3.5 ppm) are still clearly distinguishable at 10 °C. This provides evidence of a substantial remaining hydration of the polyzwitterion well below CP_UCST_. The somewhat elevated transition temperature CP_UCST_ in heavy water (D_2_O) compared to light water (H_2_O) (*cf.* Table 3) is characteristic of the thermoresponsive behavior of many poly(sulfobetaine)s, including PSBE, but is not yet understood [75,76]. 

In contrast, the signal characteristics for the PEG block, which are highlighted in yellow, are rather narrow in all spectra, and do not lose intensity at high temperatures (i.e., above CP_LCST_). This suggests that the PEG is always rather mobile, pointing to a still extensive hydration of the block above the apparent LCST-type transition. Also, the lack of a notable broadening of the various signal groups at high temperatures may indicate that the interaction between the two blocks is not very strong. This would be consistent with the above hypothesized need for matching block lengths. Although remaining speculative at present, a possible model for the two-step, thermal self-assembly is sketched in Scheme 2. 

**Figure 7:**
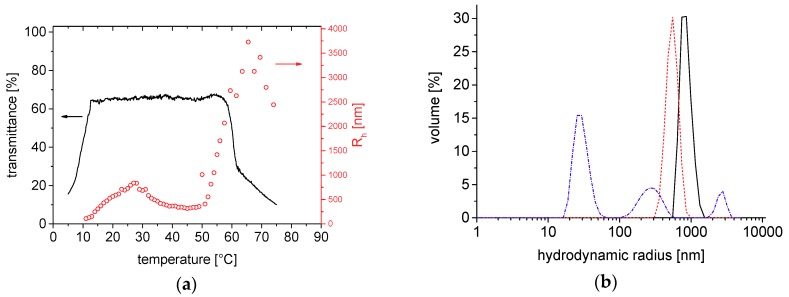
Temperature dependent study (cooling run) of 3.0 g·L^−1^ solutions in NSS of ZPE-based block polymer PEG-*b*-PZPE-1: (**a**) by turbidimetry (cooling run); values for the apparent average hydrodynamic radius *R*_h_ are also shown (**O**); (**b**) distributions of the hydrodynamic radii at 11 °C (**-·-·-·-**), 35 °C (**- - - - -**), and 75 °C (**—**).

In contrast to the studies in pure aqueous solution, only polymers incorporating the sulfabetaine ZPE showed an UCST-transition in NSS (Table 3), as illustrated in Figure 7. For the sulfabetaine block copolymer PEG-*b*-PZPE-1, which is not thermo-responsive in pure water, the temperature dependent turbidity shows not only an UCST-transition at CP_UCST_ ~12 °C in NSS, but additionally the appearance of an LCST-transition at about 55 °C, i.e., a “schizophrenic” thermoresponsive behavior similar to that found for PEG-*b*-PSBE-1 in pure water. As the values of CP_UCST_ and CP_LCST_ are further apart than in the latter system, the transitions appear to be more distinct, and the transmittance value shows a plateau (Figure 7a). Nevertheless, this plateau value is around 60% of transmittance only, so the solution looks hazy. As discussed above for PEG-*b*-PSBE-1, this observation matches previous findings for other “schizophrenic” nonionic-zwitterionic diblock copolymers, and was explained by concentration fluctuations. Here it seems also to indicate some loose aggregation of the polymers even in the intermediate temperature range, taking the DLS data into account (Figure 7a,b). Similar to the behavior of PEG-*b*-PSBE-1 discussed above, in the intermediate temperatures range of 13–50 °C, aggregates can be seen. The still substantial transmittance of the solution suggests that the rather large aggregates are loose with a high water content and thus a low refractive index contrast. The steep decrease of transmittance upon cooling below 12 °C is a result of the transformation of these loose aggregates into a mixture of more dense small objects, possibly individual macromolecules, and very large, μm-sized aggregates. At temperatures above 50 °C, the increased turbidity is the consequence of the growth and possibly also the densification of the intermediate loose aggregates.

For the statistical block copolymer PEG-*b*-P(SPE-*co*-ZPE)-1, in which about 20 mol % of ZPE units replace SPE in the zwitterionic block (*cf.* Table 2), the thermoresponsive behavior resembles closely that of the block copolymer series PEG-*b*-PSPE, namely PEG-*b*-PSPE-1 to PEG-*b*-PSPE-6. In pure aqueous solution, CP_UCST_ was about 40 °C and thus in an advantageous temperature range, but despite the notable content of ZPE units, the UCST transition is still completely suppressed in NSS. 

**Figure 8:**
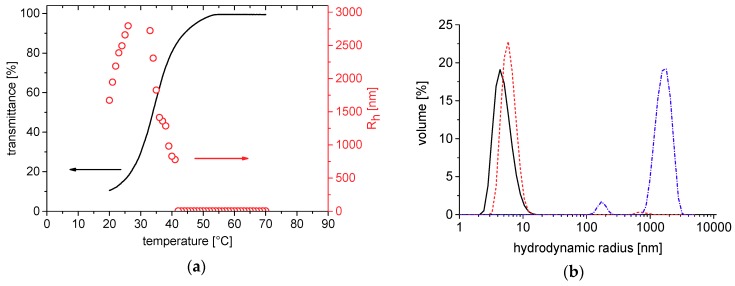
Temperature dependent study of 3.0 g·L^−1^ solutions of statistical block copolymer PEG-*b*-P(SPE-*co*-ZPE)-2 in NSS: (**a**) by turbidimetry (cooling run); values for the apparent average hydrodynamic radius *R*_h_ are also shown. (**O**); (**b**) distributions of the hydrodynamic radii at 20 °C (**-·-·-·-**), 41 °C (**- - - - -**), and 70 °C (**—**).

When the ZPE content in the statistical copolymer block is further increased, however, the thermoresponsive behavior seems particularly interesting. For the statistical block copolymer PEG-*b*-P(SPE-*co*-ZPE)-2, which contains about equal amounts of SPE and ZPE monomers in the zwitterionic block, an UCST transition is observed in pure water, even though the CP_UCST_ of about 80 °C seems impractically high for most potential uses. However, PEG-*b*-P(SPE-*co*-ZPE)-2 also presents an UCST-transition in NSS, with a CP_UCST_ of about 45 °C (Figure 8). This is in the physiologically most interesting temperature window of 30–50 °C. 

The thermal transition indicated by the change of transmittance (Figure 8a) between an individually dissolved and an aggregated state is corroborated by temperature-dependent DLS studies. At elevated temperatures, the hydrodynamic radius is well below 10 nm, thus pointing to individually dissolved macromolecules (Figure 8a,b). When going below CP_UCST_, large aggregates are formed, suggesting the formation of clusters of micelles. The transition is rather sharp, producing at 41 °C—i.e., just below CP_UCST_—intermediately a bimodal size distribution with individually dissolved macromolecules and some coexisting large aggregates (Figure 8b). Accordingly, this and closely-related block copolymer structures are attractive candidates for “smart” polymeric amphiphiles aimed at uses in the cosmetic or biomedical field, etc. The UCST-type thermoresponsivity is operative at physiologically-relevant salinity, the CP_UCST_ of the statistical thermoresponsive copolymer block may be easily fine-tuned by the block’s composition and/or molar mass, and the combination of nonionic PEG and the zwitterionic block promises a high degree of biocompatibility. 

### 3.3. Solubilization Attempts of Hydrophobic Guest by Thermoresponsive Polyethylene Glycol-Polyzwitterion Diblock Copolymers in Water and in Normal Saline Solution

**Figure 9:**
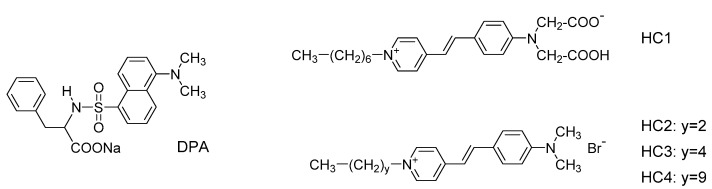
Chemical structure of the solvatochromic fluorescent dyes used in solubilization studies.

The solubilization of sparingly soluble or water-insoluble hydrophobic guest molecules (“cargo”) by amphiphilic block copolymers is well established [1,5,92]. Also, stimulus-responsive solubilization, i.e., the induced uptake and release of guest molecules has been demonstrated by numerous examples, typically exploiting a pH- or, in the case of thermoresponsive polymers, an LCST transition as the trigger [13,15,25]. In contrast, little is known about the induced uptake and release of guest molecules triggered by an UCST-transition. The scarce reports have typically employed solvatochromic dyes as model cargos. The findings suggest rather more gradually modulated release rates than their sudden increase when passing through the phase transition [56,60,77]. Moreover, it has not become clear to which extent polyzwitterions can efficiently solubilize hydrophobic guests in aqueous media, even if they are in the collapsed state above their cloud point CP_UCST_. Among the few studies on the topic available, remarkable failures to incorporate neutral solvatochromic dyes have been reported [32,65]. This may suggest that the partition of hydrophobic compounds between an aqueous and a zwitterionic phase (with its high content of ionic entities) is not advantageous, in contrast to non-ionic polymers above their LCST transition. It may be possible that in the (also few) cases reporting successful solubilization by the zwitterionic blocks of polymers [60,77,93,94], the hydrophobic fragments contained in these blocks were responsible for the dye uptake, rather than the zwitterionic moieties. It seems, at least, that complementary electrostatic interactions support the solubilization of ionic hydrophobic guests [95,96,97], in analogy to the homogeneous mixing in the bulk of up to stoichiometric amounts of certain organic dyes by polyzwitterions [98,99]. Therefore, we undertook first experiments using solvatochromic dyes (Figure 9) to verify the possibility of UCST-triggered solubilization by our zwitterionic block copolymers. For these, we selected PEG-*b*-P(SPE-*co*-ZPE)-2 by virtue of its well positioned CP_UCST_ around body temperature in NSS.

In these orienting studies, we screened the solubilization of a set of anionic (DPA, HC1) and cationic (HC2, HC3, HC4) solvatochromic fluorescent dyes (Figure 9), that are known to be solubilized by classic low- and high-molar-mass micellar systems, and which show a marked change of their emission properties (such as quantum yield or emission wavelengths) when present in an organic or an aqueous environment. 

However, neither the anionic DPA, nor the cationic HC2-HC4, exhibited convincing signs of successful uptake by PEG-*b*-P(SPE-*co*-ZPE)-2. Except for the standard moderate decrease of fluorescence intensity with increasing temperature, no meaningful difference in their individual fluorescence characteristics was observed at low and high temperatures, namely below or above CP_UCST_, either in the presence or in the absence of PEG-*b*-P(SPE-*co*-ZPE)-2. Accordingly, no solubilization could be achieved, precluding controlled delivery experiments. These findings corroborate previous studies that revealed the inability of other thermoresponsive polysulfobetaine block copolymers to accommodate polar dyes in the collapsed state [32,65]. Apparently, the interior of the collapsed but still water-swollen polymer chains is not sufficiently lipophilic for an effective partitioning of the dyes in the polymer-rich microphase compared to the matrix solvent phase. This makes a major difference to LCST-based responsive delivery systems. 

**Figure 10:**
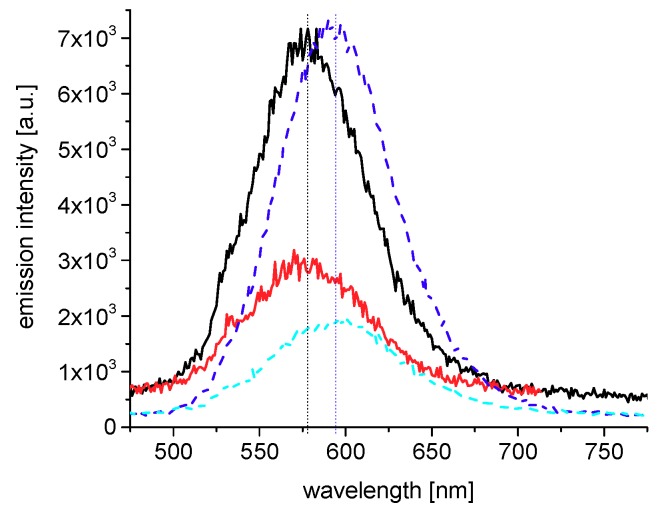
Temperature-dependent solubilization studies of the solvatochromic fluorescent dye HC1 in NSS by the statistical block copolymer PEG-*b*-P(SPE-*co*-ZPE)-2: (**—**) = emission at 25 °C in the presence of PEG-*b*-P(SPE-*co*-ZPE)-2, (**—**) = emission at 75 °C in the presence of PEG-*b*-P(SPE-*co*-ZPE)-2, (**- - - -**) = emission at 25 °C in the absence of PEG-*b*-P(SPE-*co*-ZPE)-2 (control 1), (**- - - -**) = emission at 75 °C in the absence of PEG-*b*-P(SPE-*co*-ZPE)-2 (control 2).

The situation was different for the anionic/ampholytic fluorophore HC1 in NSS. Here, the emission maximum of the dye in the presence of PEG-*b*-P(SPE-*co*-ZPE)-2 was significantly blue-shifted to 578 nm compared to the control sample of HC1 in NSS without the copolymer present, where the emission maximum was at 594 nm (Figure 10). In a simplistic interpretation, the blue-shift should be indicative of an increased polarity felt by the dye in the presence of the zwitterionic copolymer. However, such a direct correlation of the wavelength of the emission maximum and the polarity of the fluorophore’s environment was shown to be only valid within the series of linear alcohol homologues [83]. Notwithstanding the presently unclear structural implications of the blue shift observed, the shift indicates an interaction of the dye with the polymer. Yet, although the spectra differ markedly in the presence and absence of the copolymer, they do not exhibit a meaningful evolution with increasing temperature for a given solution, i.e., with or without the copolymer. We only observed the expected moderate decrease of emission intensity, which is not conclusive with respect to a polarity change of the dye’s environment. However, no spectral shift was noted upon heating above CP_UCST_. This finding could imply two scenarios, either the dye is bound by the polysulfobetaine block independent of its solution state, or the dye binds to the PEG block, which is not thermoresponsive in NSS. Whatever the explanation, hemicyanine HC1 does not experience the aspired UCST-transition-triggered controlled uptake and release scenario, which might have been expected in the presence of thermoresponsive PEG-*b*-P(SPE-*co*-ZPE)-2. 

Hence, follow-up studies might explore alternative dye structures with respect to their ability to be solubilized by thermoresponsive zwitterionic block copolymers. Alternatively, new polymer designs that incorporate more hydrophobic fragments into the polyzwitterion block might be considered, in order to favor the solubilization of standard hydrophobic cargo molecules. As the latter approach will affect the UCST-type transition temperatures, a cumbersome optimization of the polymer structure seems to be necessary for this strategy. 

## 4. Conclusions

Nonionic-zwitterionic diblock copolymers were successfully synthesized via ATRP of sulfobetaine and sulfabetaine methacrylates, employing a PEG5000 macro initiator. These complex polyzwitterions exhibit thermoresponsive behavior of the UCST type in aqueous media that can be tuned not only by the chemical structure of the monomers and the degree of polymerization, but also by copolymerizing sulfobetaines with sulfabetaines. This is particularly helpful when implementing UCST behavior both in pure water as well as in normal saline solution (NSS) within the physiologically interesting temperature window of 30–50 °C. While the rather easily available sulfobetaine monomers SPE and SBE do not provide homopolymer blocks that are still operative as thermoresponsive elements in NSS, the UCST transitions of the structurally analogous sulfabetaine polymers are exceedingly high in pure water. Thus, copolymerization of the two zwitterionic monomer classes is an attractive and variable strategy to implement thermoresponsive behavior at low as well as high salinity. Exploratory experiments on the UCST-transition triggered the encapsulation and release of a set of solvatochromic fluorescent dyes, revealed difficulties in using polyzwitterion blocks for the solubilization of organic molecules as cargos in aqueous media. This seems to be problematic even if the dyes bear ionic moieties for an enhanced interaction with the zwitterionic moieties. Only in one case could a solvatochromic effect in the emission spectra due to the presence of a zwitterionic block copolymer be observed, but its origin is still unclear. 

Although disappointing with respect to the potential applications of the block copolymers prepared, the low affinity of polyzwitterions, even in the collapsed state, toward many functional organic compounds seems a most interesting feature. In contrast to the widely reported uptake of standard active agents by nonionic polymers experiencing an LCST transition in the collapsed state, this particularity of polyzwitterions may enable the selective uptake, transport and delivery of specifically-designed cargos in mixtures where “classic” lipophilic aggregates are present (as in most biological environments), and which risk to interfere with the transport of standard organic solubilizates. Moreover, the low affinity toward most hydrophobic organic substances may also be one reason for the low fouling properties of many surfaces functionalized with polyzwitterions. 

## Figures and Tables

**Table 1:** Analytical data of block copolymers mPEG_114_-*b*-PX_n_ obtained by ATRP in solvent TFE using mPEG-Br as macroinitiator, CuBr/2,2-bipyridyl as catalyst system at 60 °C/24 h, and reagents engaged.

Polymer sample	Monomer X	[M]:[I] ^a^	Conv. [%]	Yield [%]	DP_n_	*M*_n_^theo^ [kg·mol^−1^] ^b^	*M*_n_^NMR^ [kg·mol^−1^] ^c^	*M*_n_^app^ [kg·mol^−1^] ^d^	Ð
PEG-*b*-PSPE-1	SPE	100:1	83	85	84	28	29	45	1.3
PEG-*b*-PSPE-2 ^e^	SPE	100:1	86	74	74	29	26	35	1.7
PEG-*b*-PSPE-3 ^f^	SPE	30:1	~100	70	22	13	11	20	1.4
PEG-*b*-PSPE-4 ^f^	SPE	50:1	~100	80	38	18	16	19	1.6
PEG-*b*-PSPE-5 ^f^	SPE	100:1	~100	83	81	32	28	30	1.7
PEG-*b*-PSPE-6 ^f^	SPE	200:1	~100	73	137	58	43	36	2.1
PEG-*b*-PSBE-1	SBE	100:1	73	70	70	26	26	42	1.4
PEG-*b*-PSBE-2	SBE	200:1	~100	91	183	60	59	71	1.3
PEG-*b*-PZPE-1	ZPE	100:1	n.d. ^g^	64	58	23	22	53	1.7

^a^ ratio of monomer to macroinitiator mPEG-Br; ^b^ calculated from the ratio [M]:[I] and the conversion; ^c^ via ^1^H NMR by comparing the integrals of the signals of the PEG-block to the signals of the polyzwitterion block; ^d^ SEC in HFIP containing 50 mM of CF_3_COONa, calibration with PMMA (Figure S2); ^e^ HMTETA used as ligand instead of bpy; ^f^ in CH_3_OH/H_2_O (2/3 *v*/*v*), 5 h at 23 °C, no ascorbate added; ^g^ not determined.

**Table 2:** Analytical data of statistical copolymers of SPE and ZPE, and of statistical block copolymers mPEG_114_-*b*-P(SPE-*co*-ZPE)_n_ obtained by ATRP in solvent TFE using EBiB as low molar mass and mPEG-Br as as macroinitiator, respectively, and CuBr/2,2-bipyridyl as catalyst system at 60 °C/24 h, and reagents engaged.

Polymer sample	[SPE]:[ZPE] feed	[M]:[I] ^a^	Conv. [%]	Yield [%]	*M*_n_^theo b^ [kg·mol^−1^]	*M*_n_^app c^ [kg·mol^−1^]	Ð	Q ^d^
PSPE-1 ^e^	100:0	100:1	60	43	16	26	1.6	0
PSPE-2 ^e,f^	100:0	100:1	96	72	27	41	1.6	0
PSPE-3 ^e,g^	100:0	100:1	96	72	27	32	1.5	0
PZPE-1 ^e^	0:100	50:1	80	84	12	19	1.3	100
PZPE-2 ^e^	0:100	100:1	60	n.d.	18	28	1.4	100
PZPE-3 ^e^	0:100	200:1	97	88	57	77	1.9	100
PSPE-*co*-ZPE-1 ^e^	80:20	100:1	85	80	24	18	1.4	18
PSPE-*co*-ZPE-2 ^e^	50:50	100:1	90	88	26	23	1.5	48
PSPE-*co*-ZPE-3 ^e^	20:80	100:1	95	88	28	25	1.5	76
PEG-*b*-P(SPE-*co*-ZPE)-1 ^h^	80:20	100:1	~100	84	32	30	1.8	18
PEG-*b*-P(SPE-*co*-ZPE)-2 ^h^	50:50	100:1	~100	90	32	35	2.5	48

^a^ ratio of monomer to initiator; ^b^ calculated from the ratio [M]:[I] and the conversion assuming that the ratio [SPE]/[ZPE] in the feed is preserved in the copolymer; ^c^ SEC in HFIP containing 50 mM of CF_3_COONa, calibration with PMMA (Figure S2); ^d^ content of ZPE in polyzwitterion block in mol %, calculated from the ^1^H NMR spectra in 0.5 M NaCl in D_2_O; ^e^ using initiator EBiB; ^f^ using CuBr/PMDETA as catalyst system; ^g^ using CuBr/HMTETA as catalyst system; ^h^ using macroinitiator mPEG-Br and solvent CH_3_OH/H_2_O (2/3 *v*/*v*), 5 h at 23 °C in, no ascorbate added.

**Table 3:** Cloud points CP_UCST_ and CP_LCST_ of 0.3 or 3 wt % solutions of sulfobetaine homo- and copolymers in H_2_O and in 0.154 M aqueous NaCl (NSS), determined during cooling runs.

Polymer sample	Concn. g·L^−1^	CP_UCST_ in H_2_O [°C]	CP_LCST_ in H_2_O [°C]	CP_UCST_ in NSS [°C]	CP_LCST_ in NSS [°C]
PSPE-1	30.0	23	-	<0	-
PSPE-2	30.0	18	-	<0	-
PSPE-3	30.0	22	-	<0	-
PZPE-1	3.00	>100	-	47	-
PZPE-2	3.00	>100	-	60	-
PZPE-3	3.00	>100	-	~100	-
PSPE-*co*-ZPE-1	3.00	20	-	<0	-
PSPE-*co*-ZPE-2	3.00	62	-	22	-
PSPE-*co*-ZPE-3	3.00	70	-	42	-
PEG-*b*-PSPE-1	30.0	54	-	<0	-
PEG-*b*-PSPE-2	30.0	48	-	<0	-
PEG-*b*-PSPE-3	30.0	39	-	<0	-
PEG-*b*-PSPE-4	30.0	45	-	<0	-
PEG-*b*-PSPE-5	30.0	65	-	<0	-
PEG-*b*-PSPE-6	30.0	50	-	<0	-
PEG-*b*-PSBE-1	30.0	30	45	<0	20
PEG-*b*-PSBE-2	30.0	≥80	-	<0	-
PEG-*b*-PZPE-1	3.00	>100	-	12	55
PEG-*b*-P(SPE-*co*-ZPE)-1	3.00	43	-	<0	-
PEG-*b*-P(SPE-*co*-ZPE)-2	3.00	~80	-	45	-

## Supplementary Materials

The following are available online at http://www.mdpi.com/2073-4360/10/3/325/s1, Figure S1: Scheme of polymer synthesis, Figure S2: SEC elugrams of the polymers, Figure S3: Temperature dependent DLS studies of aqueous solutions of block copolymer PEG-*b*-PSBE-1.
