# Supplementary material for: Exploring Poly(ethylene glycol)-Polyzwitterion Diblock Copolymers as Biocompatible Smart Macrosurfactants Featuring UCST-Phase Behavior in Normal Saline Solution
Source: Polymers (Basel). 2018 Mar 15;10(3):325. doi: 10.3390/polym10030325 (PMC6414896; doi:10.3390/polym10030325)
Supplement: Supplementary file 1 [file polymers-10-00325-s001.pdf]

# Supplementary Material

# Exploring Poly(ethylene glycol)-Polyzwitterion Diblock Copolymers as Biocompatible Smart Macrosurfactants Featuring UCST-Phase Behavior in Normal Saline Solution

**Noverra M. Nizardo [1], Dirk Schanzenbach, [1] Eric Schönemann [1] and André Laschewsky [1,2*]**

[1] University of Potsdam, Institute of Chemistry, Karl-Liebknecht-Str. 24-25, D-14476 Potsdam-Golm, Germany; e-mails: nizardo@uni-potsdam.de (N.M.N.), .), dschanz@uni-potsdam.de (D.S.), eschoenemann@uni-potsdam.de (E.S.), laschews@uni-potsdam.de (A.L.)

[2] Fraunhofer Institute of Applied Polymer Research IAP Geiselberg-Str. 69, D-14476 Potsdam-Golm, Germany; e-Mail: andre.laschewsky@iap.fraunhofer.de (A.L.)

***** Author to whom correspondence should be addressed; e-Mail: laschews@uni-potsdam.de (A.L.); Fax: +49-331-997-5036. ORCID number 0000-0003-2443-886X.

## 1. Polymer synthesis

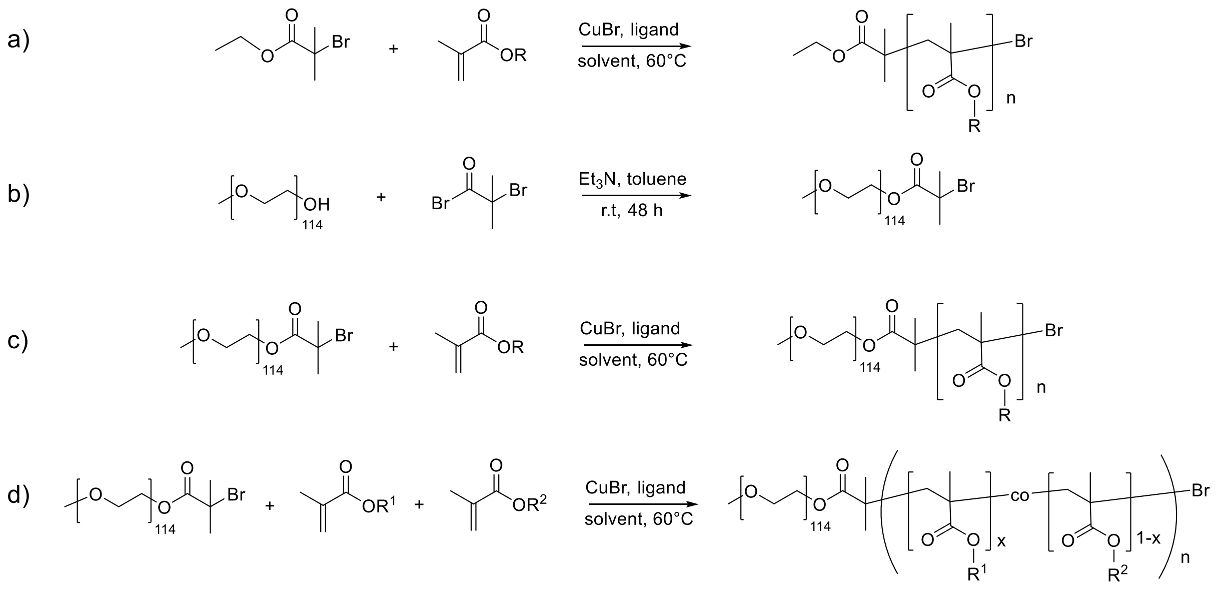


**Figure S1.** Synthetic pathways to : (a): homopolymers; (b) macroinitator; (c) block copolymers; (d) statistical block copolymers.

## 2. Polymer characterization

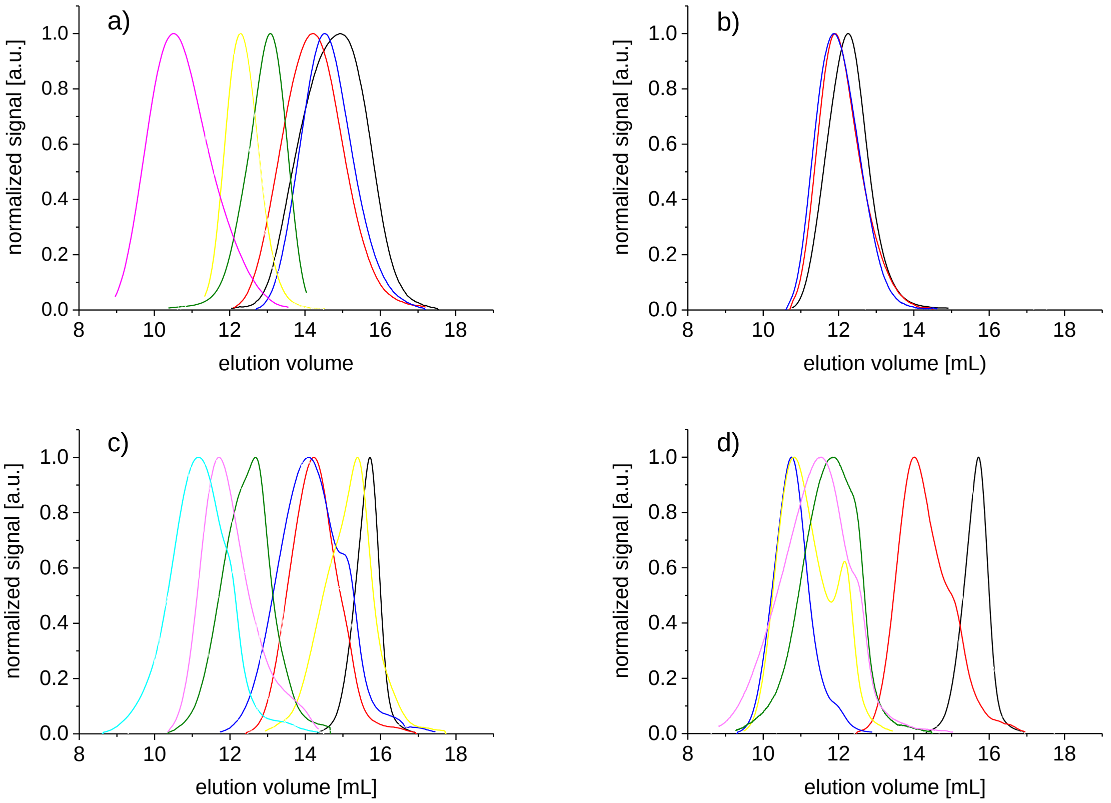


**Figure S2.** SEC elugrams of the polymer studied (eluent: hexafluoroisopranol (HFIP) containing 50 mM of sodium trifluoroacetate, calibration with narrowly distributed poly(methyl methacrylate) standards.

a) homopolymers: (——) = **SPE-1**, (——) = **SPE-2**, (——) = **SPE-3**, (——) = **ZPE-1**, (——) = **ZPE-2**, (——) = **ZPE-3**;

b) statistical copolymers: (——) = **PSPE-*co*-ZPE-1**, (——) = **PSPE-*co*-ZPE-2**, (——) = **PSPE-*co*-ZPE-3;**

c) macroinitiator **mPEG-Br** (——), and block copolymers: (——) = **PEG-*b*-PSPE-1**, (——) = **PEG-*b*-PSPE-2**, (——) = **PEG-*b*-PSPE-3**, (——) = **PEG-*b*-PSPE-4**, (——) = **PEG-*b*-PSPE-5**; (——) = **PEG-*b* -PSPE-6;**

d) macroinitiator **mPEG-Br** (——) =, block copolymers: (——) = **PEG-*b*-PSBE-1**, (——) = **PEG-*b*-PSBE-2**, and statistical block copolymers: (——) = **PEG-*b*-PZPE-1**, (——) = **PEG-*b*-P(SPE-*co*-ZPE)-1**, (——) = **PEG-*b*-P(SPE-*co*-ZPE)-2.**

## 3. Polymer studies in aqueous solution

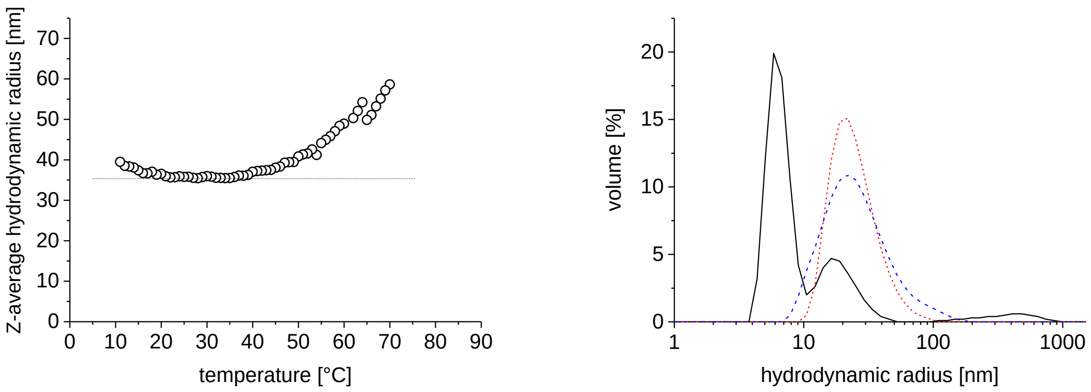


(a) (b)

**Figure S3.** Temperature dependent evolution of the hydrodynamic radius of solutions of block copolymer PEG-*b*-PSBE-1 in water at 30.0 g $L^{-1}$: (a) average hydrodynamic radius $R_h$ (cooling run, the dotted line is meant as a guide to the eye); (b) distributions of the hydrodynamic radii at 20 °C ( -··-··-), 40 °C (- - - - - ), and 70 °C ( ——— ).
